# Effect of Lutein on Ocular Goblet Cell, IFN-γ, and IL-17 Concentration in Dry Eye-Induced Mice Model

**DOI:** 10.7759/cureus.42009

**Published:** 2023-07-17

**Authors:** Giovani Faustine, Ani R Prijanti, Heri Wibowo

**Affiliations:** 1 Immunology, Universitas Indonesia, Jakarta, IDN; 2 Biochemistry and Molecular Biology, Universitas Indonesia, Jakarta, IDN; 3 Parasitology, Universitas Indonesia, Jakarta, IDN

**Keywords:** lutein, il-17, ifn-γ, goblet cell, dry eye

## Abstract

Introduction

Dry eye disease affects a substantial number of individuals globally and significantly impacts their quality of life and productivity. Understanding the underlying mechanisms and managing dry eye disease poses substantial challenges. Recent research has highlighted the involvement of various inflammatory mediators in the pathogenesis of dry eye disease, including the cytokines interferon (IFN)-γ and interleukin (IL)-17. Activation of stress signaling pathways and residential immune cells on the ocular epithelial surface ignites epithelial changes, destabilizes tear film, amplifies inflammation and creates an endless loop. Lutein is a β-carotenoid antioxidant which has been proven to be beneficial in many ocular diseases due to its protective and anti-inflammatory effect induced by various stimulators. Lutein also acts as a direct and indirect antioxidant agent, suppressing oxidative stress and mitigating oxidative damage. The purpose of this research is to investigate the potential therapeutic effects of lutein in a mouse model of dry eye, aiming to elucidate its impact on ocular manifestation, goblet cells count, IFN-γ and IL-17 level.

Methods

Desiccating stress was induced in C57BL/6 mice. In a separate group, lutein was administered subcutaneously on a daily basis throughout the experimental period. Clinical manifestations of dry eye, including ocular surface changes, were documented photographically. Goblet cell concentration was assessed through Periodic Acid-Schiff (PAS) staining, and the levels of IFN-γ and IL-17 were measured using enzyme-linked immunosorbent assay (ELISA). Data obtained from these assessments were compared between the experimental groups to determine the potential effects of lutein on dry eye pathology and cytokine levels.

Results

Significant differences were observed in clinical observations and goblet cell concentrations among the groups; however, no statistically significant differences were found in the levels of IFN-γ and IL-17 between the groups. The untreated group exhibited significantly higher opacities and irregularities compared to the lutein-treated group, whereas the mean goblet cell count was highest in the lutein-treated group.

Conclusion

Lutein administration improves clinical observations and goblet cell concentrations in a mouse model of dry eye. The treated group exhibited improved ocular surface integrity, but no significant differences in the tested cytokine levels were observed. These findings suggest that lutein supplementation could be a promising therapeutic option for managing dry eye disease. Further research is needed to understand the underlying mechanisms and long-term effects of lutein in dry eye management.

## Introduction

Keratoconjunctivitis sicca, commonly known as dry eye, is a disruption in ocular surface and tear film [1]. It is often a multifactorial disease that causes irritation and discomfort, two of the most frequent chief complaints found in outpatients. Tear instability and hyperosmolarity will activate stress signaling pathway in the ocular epithelial surface where inflammatory responses and epithelial changes will impair the tear film, and escalate the inflammatory process, thus producing an incessant inflammatory cycle.

Around 5-34% of people worldwide have dry eye disease [1]. Stapleton et al. stated the number even reaches 50-75% in adults above 40 years, predominated with women [2]. This wide range of prevalence is influenced by a variety of population studies, geographical features, methods, and different standards in defining the disease.

Nichols et al. stated that dry eye caused a 0.36% loss of working time and presenteeism, decreased work productivity, and other work-related activities [3]. The effect on work performance and overall productivity is also substantial where there is a 29% decline than normal. Daily activity performance was also reduced by as much as 30% [3].

Acute desiccation stress that occurs on the ocular surface will activate a number of signaling pathways on ocular surface immune residents. Production of inflammatory mediators such as tumor necrosis factor (TNF)-α, IL-1, IL-6, IL-8, IL-17, IL-23, IFN-γ, chemokines and matrix metalloproteinases (MMPs), vascular endothelial growth factor (VEGF) and its receptors will attract antigen-presenting cells and lymphocytes (T helper 1, T helper 17) towards ocular surface causing tight-junction lysis, corneal desquamation, epithelial apoptosis, tear instability and amplifying inflammation process. The increase of IFN-γ level is also noted in dry eye patients [4]. IFN-γ has long been known to exacerbate apoptosis in dry eye-induced goblet cells in conjunctiva through dual apoptotic pathways [5], compromising their essential function of producing and secreting mucus, leading to a reduction in mucus production and compromising the ocular surface's protective barrier. IL-17 plays a role in the pathogenesis of ocular surface and corneal disease and targeting this cytokine may provide a useful treatment option in the future [6]. These signaling pathways eventually stimulate transcription factors which will elicit stronger innate and adaptive immune response.

Studies have shown that antioxidants have therapeutic potential in improving dry eye symptoms [7-9], thus becoming the reason this research is carried out. Lutein is a β-carotenoid which has been proven to have a protective effect and inhibits inflammation induced by various stimulators in vitro and in vivo [10,11]. Overall, lutein acts as an active antioxidant agent by directly scavenging and neutralizing reactive oxygen species, and as a passive antioxidant agent by enhancing the activity of endogenous antioxidants, such as superoxide dismutase or glutathione peroxidase, which help counteract oxidative stress and attenuate oxidative damage [11]. Many researchers have proven that lutein prevents occurrences and progressivity of eye diseases such as cataract, macular degeneration, diabetic retinopathy, or uveitis; inhibits various inflammatory mediators and signaling pathways; and has free radical scavenger properties. There seem to be few experiments done to analyze the effect of lutein on the progressivity of dry eye disease. This research is done to analyze the effect of lutein on clinical results, goblet cell concentration, and adaptive immune response (IFN-γ and IL-17 levels) in mice dry eye model. The widespread of commercial oral lutein supplementation as a part of daily use vitamins makes oral lutein globally achievable, thus becoming the reason we choose the oral route of administration in this research.

## Materials and methods

Animals induction and treatment group

This research protocol was approved by the Ethics Committee of the Faculty of Medicine, University of Indonesia - Cipto Mangunkusumo Hospital.

Twenty female C57BL/6 mice aged six to eight weeks old, were acclimated to the housing conditions for a period of seven days prior to the start of the experiment. All mice were ensured to start with good eye condition during the acclimation period. The mice were divided into three groups: six mice in the untreated control group, seven mice in the untreated dry eye group, and seven mice in the dry eye group supplemented with lutein. The animals were randomly assigned in these groups. The mice were housed in individual ventilated cages (IVC) with underpads used as bedding material. Each cage accommodated 3-4 mice, allowing for social interaction and minimizing stress. The desiccating model was made according to a previous study [12] with modifications. Mice were placed in an environment with less than 40% humidity and exposed to airflow 2-4m/s for 16 hours a day. Additionally, a subcutaneous injection of 0.5mg scopolamine butylbromide (Otto Pharmaceutical Industries, Bandung, Indonesia) was administrated in alternating hindquarters four times a day (at 9 AM, 12 AM, 3 PM, 6 PM). Airflow in the cage was generated using a computer fan installed in the cage. Humidity and temperature were monitored using the dehumidifying device. The mice were treated for 10 days, with the lutein group receiving a daily oral dose of 0.5mg/kg lutein (Sigma-Aldrich, St. Louis, MO, USA). Throughout the study, all mice had ad libitum access to food and water.

Clinical documentation and periodic acid Schiff staining

After 10 days of treatment, mice were euthanized by cervical dislocation. Each eye was photographed, and morphological changes were observed. Opacities seen were scaled based on the modified Fantes Haze Scale [13] to assess the severity of ocular surface irregularities. The adnexa and ocular tissues were then excised for further analysis. Segments of the bulbar and tarsal conjunctiva were subjected to periodic acid-Schiff (PAS) staining. The density of conjunctival goblet cells seen was quantified by counting the number of goblet cells per field of view using a binocular microscope. Five fields of view were analyzed per eye to ensure a representative assessment of goblet cell density.

Enzyme-linked immunosorbent assay

The ocular tissues from each group of mice were homogenized using a homogenizing machine. The resulting homogenates were centrifuged to obtain the supernatants. The concentrations of IL-17 and IFN-γ in the supernatants were measured using an ELISA Kit specifically designed for IL-17 and IFN-γ (MyBioSource, San Diego, CA, USA). The measurements were performed according to the protocol provided with the kit. The concentrations of IL-17 and IFN-γ were expressed in picograms per gram of protein.

Statistics

We used SPSS 26 software (IBM Corp., Armonk, NY, USA) throughout the statistical analysis. Haze scale measurements across different groups underwent analysis using Chi-Square, Fisher's Exact test. Goblet cell concentration data between groups were analyzed using One Way ANOVA. Post-hoc tests were performed on goblet cell concentration data to determine specific group differences. IFN-γ and IL-17 data were analyzed using the Kruskal-Wallis test as these variables did not meet the assumptions of normality. Statistical significance was set at p < 0.05. Both the right and left eyes were analyzed separately to ensure a comprehensive evaluation of the results.

## Results

Clinical findings of ocular surface

**Figure 1:**
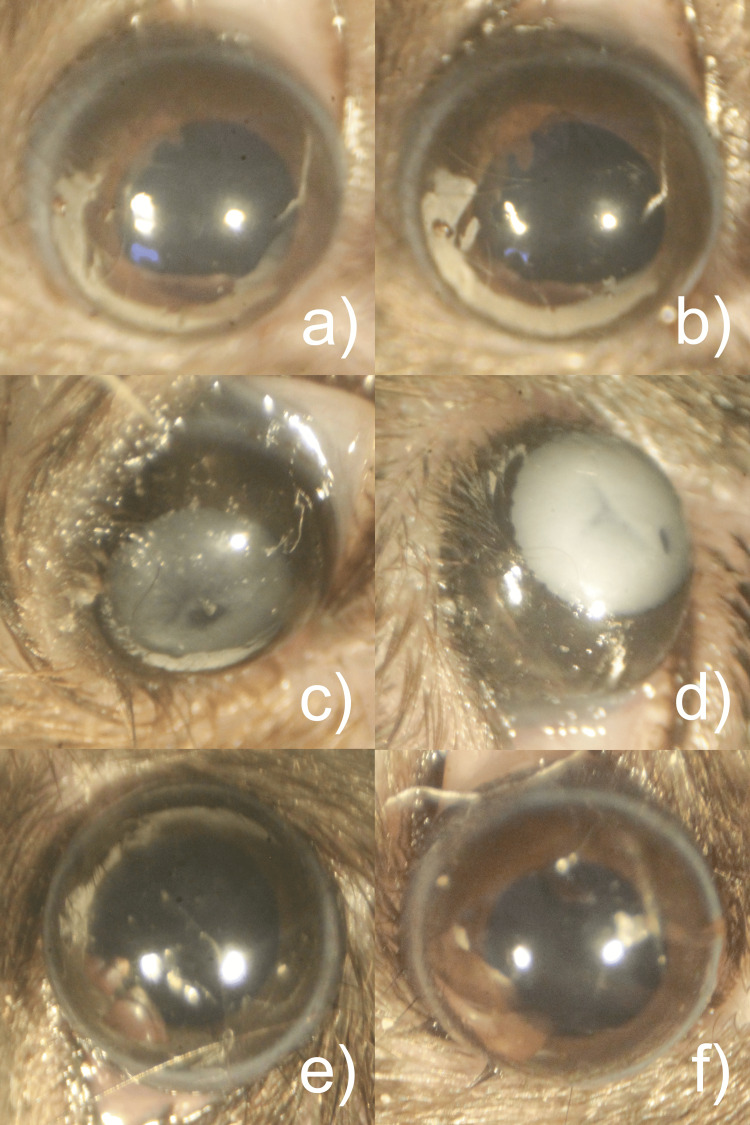
Clinical ocular appearance photograph a) and b): control group right and left; c) and d): untreated group left and right; e) and f): lutein-treated group left and right.

No ocular surface changes were observed in both the control group and the lutein-treated group. However, there were scars and opacities observed with varying degrees on the corneal surface in the untreated group after the experiment as seen in Figure 1. Scales of opacities in the untreated group are described in Table 1. There were significant differences between the control group and the lutein-treated group with the untreated group (p<0.05, respectively).

**Table 1:** Corneal ocular surface haze scale distribution among groups The table provides a breakdown of the number and percentage of mice with different scales of opacities according to the modified Fantes Haze Scale [13], as presented above.

Scale	Description	Total					
		Control		Untreated		Lutein	
		Right Eye	Left Eye	Right Eye	Left Eye	Right Eye	Left Eye
Clear	-	6 (100%)	6 (100%)	0	0	7 (100%)	7 (100%)
0	Trace haze seen with careful oblique illumination.	0	0	0	0	0	0
1	Noticeable haze not interfering with visibility of fine iris details, haze smaller than ¼ corneal area. No neovascularization.	0	0	1 (14.28%)	3 (42.85%)	0	0
2	Mild obscuration of iris details and haze larger than ¼ corneal area with or without neovascularization.	0	0	4 (57.14%)	2 (28.57%)	0	0
3	Obscuration of the iris and lens with or without neovascularization.	0	0	2 (28.57%)	2 (28.57%)	0	0
Total		6 (100%)	6 (100%)	7 (100%)	7 (100%)	7 (100%)	7 (100%)

Histological changes in goblet cells

**Figure 2:**
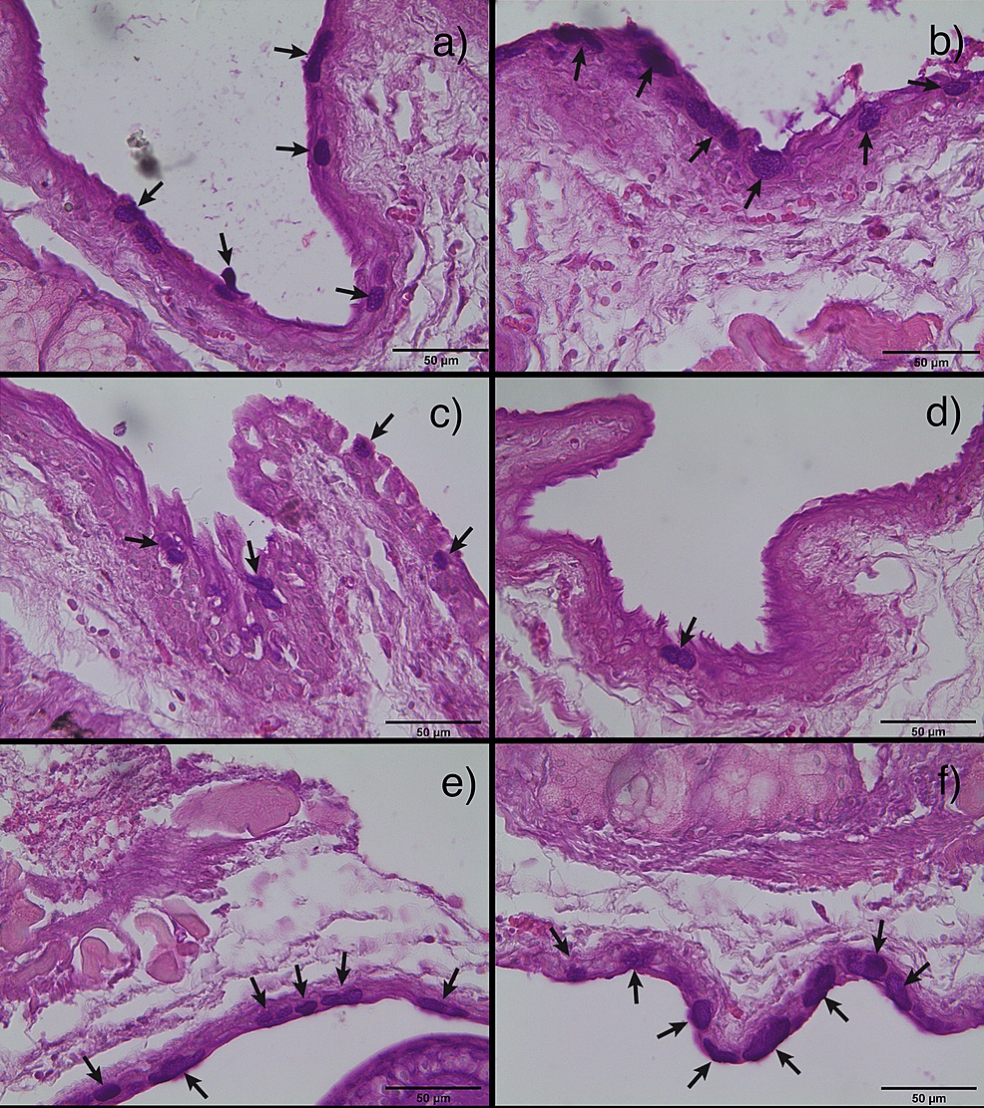
Merged images of PAS staining on conjunctival tissues Goblet cells, which exhibited a PAS-positive result (marked in black arrows), seen using 50x magnification. a) and b): control group right and left; c) and d): untreated group right and left; e) and f): lutein-treated group right and left.

**Figure 3:**
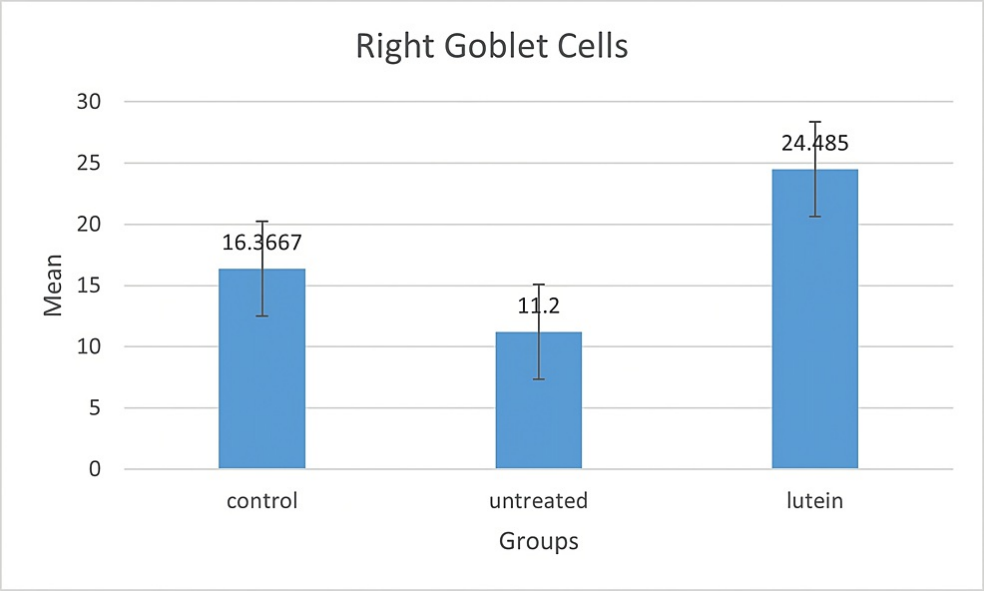
Goblet cell concentration in the right eyes The mean of goblet cell density was measured in a number of goblet cell/field. Goblet cell density in the lutein-treated group was statistically different from the other two groups. There was no significant difference between the control group and the untreated group.

**Figure 4:**
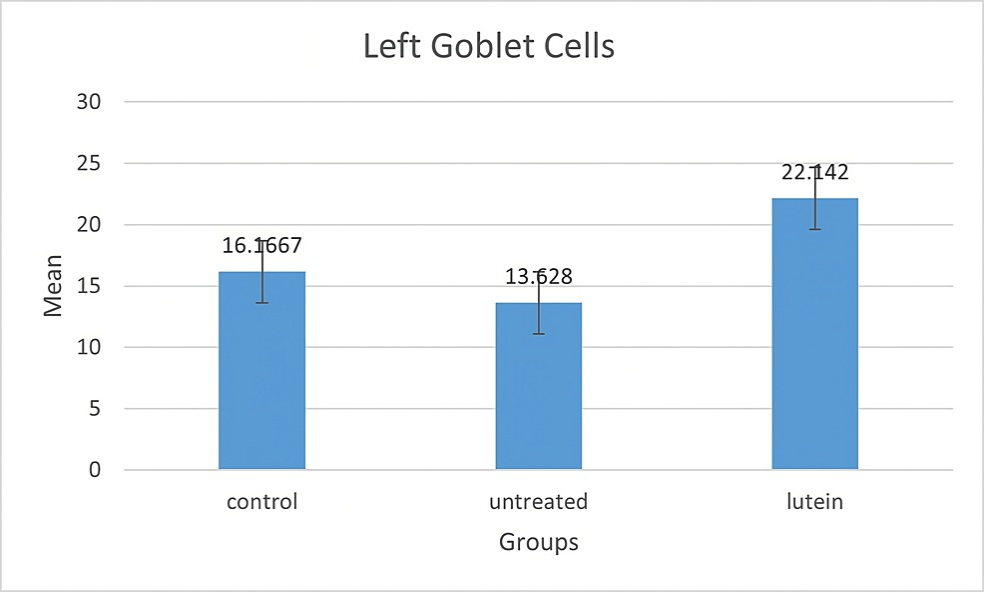
Diagram bar on goblet cell mean in the left eyes The mean of goblet cell density was measured in a number of goblet cell/field. There was a significant increase in the number of goblet cells in the lutein-treated group compared to the control group and the untreated group. No significant difference was noted between the control group and the untreated group in both eyes.

Conjunctival goblet cells were shown in PAS-positive images. The result of goblet cells stain and count could be seen in Figures 2-4. In this study, goblet cells concentration in both eyes increased significantly in the lutein-treated group (right, mean ± SD = 24.485 ± 5.417; left, mean ± SD = 22.142 ± 6.194) compared to the control group (right, mean ± SD = 16.366 ± 4.107; left, mean ± SD = 16.166 ± 2.33) (right, p = 0.005; left, p = 0.056) and the untreated group (right, mean ± SD = 11.2 ± 1.432; left, mean ± SD = 13.628 ± 2.994) (right, p < 0.001; left, p = 0.005). There was no significant difference between the control group and the untreated group in both eyes (right, p = 0.080, left, p = 0.54).

IFN-γ and IL-17 concentrations

**Figure 5:**
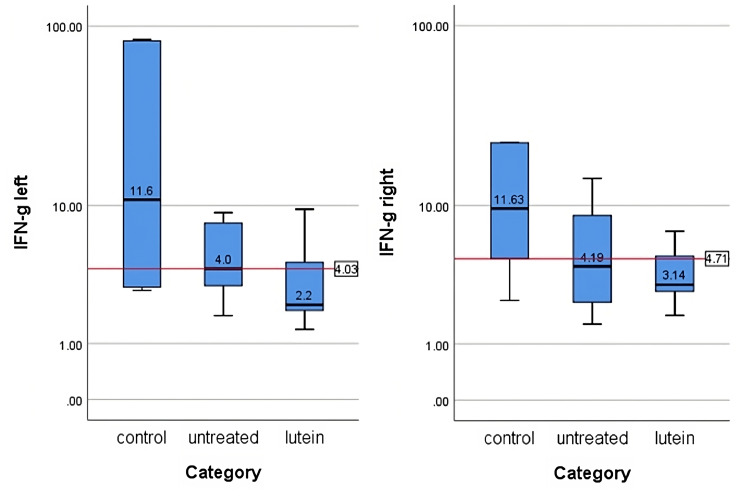
IFN-γ levels in the left and right eyes in each group The levels were measured in pg/mg protein. Median number of each group is shown in the figure. There was no significant difference in IFN-γ between all groups in both sides (p>0.05). The levels of IFN-γ were analyzed using the Kruskal-Wallis test.

**Figure 6:**
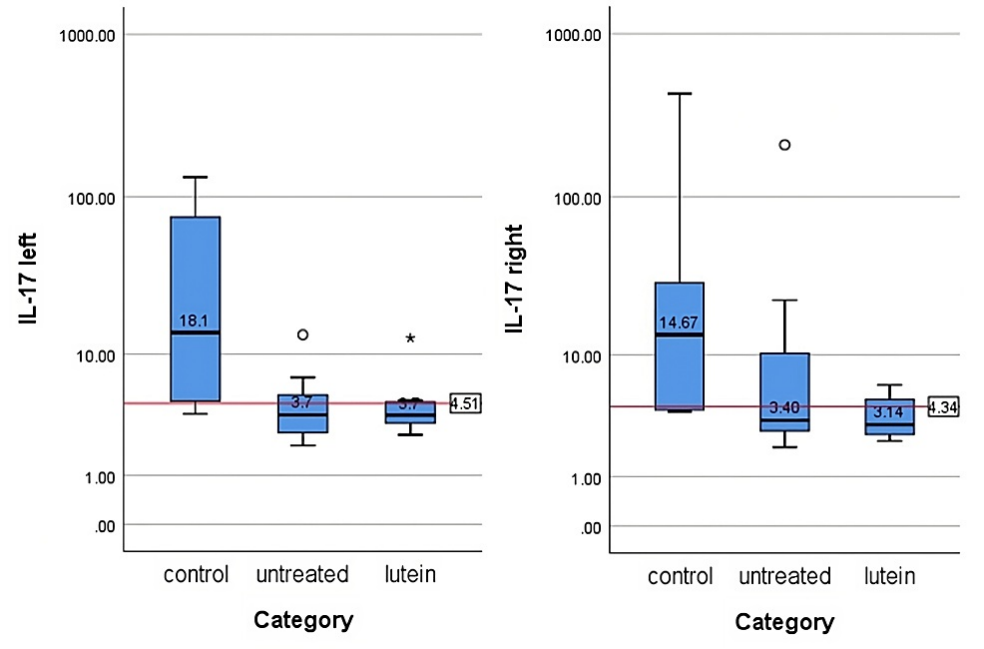
IL-17 levels in the left and right eyes in each group The levels were measured in pg/mg protein. The figure displays the median values for each group. No statistically significant difference in IL-17 levels was observed between the groups on both sides (p>0.05). The levels of IFN-γ were evaluated using the Kruskal-Wallis test.

IFN-γ and IL-17 concentrations were measured after tissues were homogenized and supernatants were collected. The protein level in each supernatant was measured and the cytokines level from ELISA was standardized with the protein level in each sample. The result was reported in pg/mg protein. The average values are summed in Table 2, Table 3, Figure 5, and Figure 6. There was no significant difference in IFN-γ (right, p = 0.27; left, p = 0.13) and IL-17 (right, p = 0.108; left, p = 0.057) between all groups in both eyes.

**Table 2:** IFN-γ levels in each group (median (min-max))

Group	Right Eye (pg/mg protein)	Left Eye (pg/mg protein)
Control	11.63 (2.41-235.63)	11.56 (2.85-84.6)
Untreated	4.19 (1.55-208.97)	4.03 (1.82-9.08)
Lutein	3.14 (1.84-7.01)	2.22 (1.38-9.5)

**Table 3:** IL-17 levels in each group (median (min-max))

Group	Right Eye (pg/mg protein)	Left Eye (pg/mg protein)
Control	14.67 (3.98-429.23)	18.13 (3.75-132.79)
Untreated	3.40 (2.02-208.76)	3.68 (2.04-13.48)
Lutein	3.14 (2.3-6.24)	3.66 (2.53-12.82)

## Discussion

In this study, 20 female mice were used to develop a desiccating-induced dry eye model based on an experiment done by Dursun et al. [12]. A combination of scopolamine injections and environmental changes were done in order to induce stress, thus creating an inflammatory response that mimics dry eye disease. All measurements between the left and right eyes were separated in order to see if there was any difference of effect between the two.

The success of dry eye induction in this trial was measured through ocular surface clinical observation, particularly using a modified Fantes Haze Scale. There was a significant clinical difference between the untreated group with control and the lutein-treated group in the study. Corneal surface opacities in the untreated group were seen in different scales at the end of the experiment while there were clear ocular surfaces in the control and lutein-treated group.

Goblet cell count is the second parameter in deciding whether there were changes in the study. Goblet cells play a crucial role in maintaining the ocular surface health by producing the protective mucus layer, which helps lubricate and protect the conjunctiva and cornea from environmental stressors, pathogens, and dryness. PAS staining of the conjunctival tissues in the untreated group had significantly lower goblet cell concentration compared with the control group. A similar trend seen on both sides suggests the effect was symmetrical. The result is in line with research done by Chen et al. where the reduction of goblet cell concentration was also seen in dry eye disease [14]. Caspase-3 expression which was induced by the IFN-γ is responsible for the conjunctival goblet cell apoptosis. The significant increase in goblet cell concentration in the lutein-treated group might indicate that lutein plays a role in the proliferation of goblet cells. Jin et al. [15] expressed that lutein has a similar inhibitory effect towards inflammation as dexamethasone when given at a higher dose. Additionally, a study by Yang et al. [16] stated that corticosteroid has the ability to reduce pro-inflammatory cytokines and improve goblet cell concentration. It can be inferred that lutein may share similar mechanisms with corticosteroids in the context of dry eye disease, potentially leading to an elevation in goblet cell concentration, and offering protective properties against the desiccating stress in mice.

IFN-γ, which is secreted exclusively by T helper 1, cytotoxic T, and natural killer cells, has important roles in regulating ocular immune responses. IFN-γ upregulates the secretion of proinflammatory cytokines including IL-1, IL-6, and TNF-α, chemokines such as IL-9, cell adhesion molecules, as well as MMP-3 and MMP-9, resulting in ocular epithelial damage [17]. De Paiva et al. [5] also indicated that desiccating stress promotes conjunctival goblet cell apoptosis and cornification through CD4+ T cell and IFN-γ infiltration into the cell. The high count of goblet cells in the lutein-treated group was also accompanied with lower IFN-γ level. Reduction of IFN-γ level supports the preservation of mucin-secreting goblet cells concentration which can provide cover from desiccation stress on the ocular surface. IL-17 also plays a role in causing stress on the corneal epithelial barrier. This barrier and corneal epithelial disruption will end in corneal surface irregularity [18]. Elevated level of IL-17 stimulates the production of MMP-3 and MMP-9 which will promote the pathogenic effects leading to ocular surface barrier disruption [18,19].

IL-17, secreted by T helper-17 cells, has the ability to increase the levels of VEGF-A, VEGF-B, VEGF-C, and VEGF-D, thereby promoting lymphangiogenesis and angiogenesis in the dry eye corneal surface [19]. No mice in this study developed neovascularization on the ocular surface. It might suggest that the death of ocular surface cells, including resident immune cells, happened before IL-17 could incite the neovascularization process. How long the time it takes for circulating IL-17 to create neovascularization in the ocular surface is still unknown.

The result of this study suggests there were no significant effects of lutein on IFN-γ and IL-17 levels. Although IFN-γ and IL-17 measurements were lower in the untreated group than the control group, there were clear signs of their involvement seen in ocular surface defects. In the lutein-treated group, the level of lutein in IFN-γ and IL-17 was as low as in the untreated group, but no damage was seen in the ocular surfaces. Lutein might play a role in increasing goblet cell count significantly and maintaining the optimal condition of the ocular surface, protecting it from inflammation process and degradation. Although statistically the ocular IFN-γ and IL-17 results in this study are contrary to the elevating level in previous studies [15,16,17-23], the consideration between clinical conditions, histological examination, and previous studies supports the theory that lutein has the capability, directly or indirectly, affecting IFN-γ and IL-17 levels. How its specific mechanism may increase goblet cell concentration and whether they are really correlated to each other need to be investigated further.

Other than reducing the level of pro-apoptosis cytokine, IFN-γ, apoptosis suppression by lutein also happens through cyclin-dependent kinase 1 [24]. This might explain why goblet cell concentration is preserved, if not increased, in the lutein-treated group. Ocular barrier damage in the lutein-treated group is also not seen clinically. Whether lutein plays a direct role in the integrity of the ocular surface or through goblet cell preservation is still to be determined. A support study also proven by Chen et al. [25] shows that lutein therapy on mice dry eye model can decrease pro-inflammatory cytokine levels and restore goblet cell number. No further research has been done to look for the correlation and mechanism between lutein supplementation and goblet cell count.

Limitations

There are many areas for potential improvement and further development in this research. While animal models provide valuable insights, they may not fully replicate the complex nature of human dry eye disease. Therefore, caution should be exercised when extrapolating the results to human patients. The limited volume of ocular tissue available for analysis posed a challenge and may impede researchers from conducting a comprehensive examination in a single attempt. Adding functional parameters such as tear production could provide a more comprehensive understanding of the lutein treatment effects. Long-term follow-up on the effects of lutein supplementation was not assessed in this research, which should be considered when planning for future experiments. A comparison of the effect between methods of delivery of lutein can also be added in the next research setup. Hopefully, this article can be an additive value in broadening our knowledge of lutein benefits in the eye inflammatory process.

## Conclusions

Our study demonstrates that lutein administration in a mouse model of dry eye resulted in significant improvements in clinical observations and goblet cell concentrations. The treated group exhibited reduced opacities and irregularities, indicating a positive effect on ocular surface integrity. However, there were no significant differences in the levels of IFN-γ and IL-17 between the groups, suggesting that lutein may not directly influence the expression of these cytokines in this experimental setting. These findings suggest that lutein supplementation holds promise as a potential therapeutic option for managing dry eye disease, specifically in terms of improving ocular surface health and goblet cell function. Further research is warranted to elucidate the underlying mechanisms and explore the long-term effects of lutein in dry eye management.
